# Prevalence of asthma in school children in rural India

**DOI:** 10.4103/1817-1737.62478

**Published:** 2010

**Authors:** Pradeepa P. Narayana, Mithra P. Prasanna, S. R. Narahari, Aggithaya M. Guruprasad

**Affiliations:** *Institute of Applied Dermatology, Kasaragod District, Kerala State, India. E-mail: pradeep.narayana@gmail.com*

Sir,

Asthma is one of the most important chronic diseases of childhood, causing substantial morbidity. Increase in the rates of hospital admission and primary care contacts for asthma in childhood has led to concern regarding prevalence or severity of increasing wheezing illness in children.[1] There is paucity of studies in India regarding asthma in children. Recent report shows wide variation (4–19%) in the prevalence of asthma in school-going children from different geographic areas in India.[2] Recognizing the problem in children is very essential, since the spectrum of presentation is variable and multiple for proper management.[3] The International Study of Asthma and Allergy in Childhood (ISAAC) developed a standardized method for describing the prevalence of asthma and other atopic disorders.[4] We carried out this study in three schools of Subramanya, a rural area in Dakshina Kannada District of Karnataka state in India. The schools in the study belonged to Government-aided and unaided managements. Hence, the study population included all the socio-economic strata. Study population included school children between 10–18 years. The total number of subjects in our study was 573. Overall, there were 55.1% males and 44.9% females. The mean age of the study population was 13.4 years (SD = 1.5). Majority of subjects belonged to 13–15 years age group in both the genders (53.5% among males and 46.5% among females).

The prevalence of ever wheezers in our study is 8.4% and current wheezers is 5.2% and among them 16.7% had one to three episodes in a year on an average. The wheezing was more prevalent among the 10–12 years age group (11.5%) compared to others. Among the 573 subjects, 18.5% had allergic rhinitis. Among them, 12% gave history of nasal block, sneezing in the absence of seasonal rhinitis and 7.3% had history of lacrimation. As shown in Table 1, the history of ever wheezing was more among males (11.7% compared to 4.3% among females). This difference was statistically significant (*P* = 0.01). The wheezing in the past 12 months was more among males among those who had a past history of wheeze (64.9% compared to 54.5% among females). This difference was not statistically significant (*P* = 0.72). The wheezing frequency was not different among the genders to attain a statistical significance (*P* = 1). Similarly, with regard to the severity of the symptoms during the attacks of Asthma, there was no gender difference.

**Table 1:** Genderwise prevalence of asthma and allergic rhinitis symptoms among the subjects

Asthma and allergic rhinitis symptoms	Males No. (%)	Females No. (%)	Grand total	*P*
History of wheezing in the past	37 (11.7)	11 (04.3)	573	0.001*
Past 12 months history of wheeze	24 (64.9)	6 (54.5)	48	0.724
Frequency of wheezing in past 12 months (1 to 3 times)	31 (83.8)	9 (81.8)	48	1.000
Unable to utter 2-3 words due to difficulty in breathing in past 12 months	17 (45.9)	2 (18.2)	48	0.161
History of diagnosed asthma	14 (43.8)	0 (0.00)	48	0.036*
Exercise induced breathlessness in past 12 months	33 (10.4)	12 (4.7)	573	0.011
Dry cough in the past 12 months (other than sunning nose and expectoration)	92 (29.1)	30 (11.7)	573	0.0001*
Nasal discharge in absence of seasonal rhinitis	79 (25.0)	27 (10.5)	573	0.0001*
Sneezing, nasal block on the absence of rhinitis in past 12 months	53 (58.2)	17 (40.5)	133	0.056
Nasal symptoms along with lacrimation and nasal discharge in past 12 months	33 (44.0)	9 (25.7)	110	0.066
History of fever due to allergy to hay	20 (6.3)	5 (2.0)	573	0.011*

* *P* value < 0.05 – statistically significant difference

The presence of dry cough in the past 12 months was significantly (*P* < 0.0001) higher among the male subjects (29.1% compared to 11.7% among females). The prevalence of nasal symptoms was more among males. In the absence of seasonal rhinitis, 25% males and 10.5% females had nasal symptoms in the form of clear nasal discharge. This difference was statistically significant (*P* < 0.0001).

The rates of exacerbation were higher in the June and July (6.3% and 5.8%, respectively). Overall, the trend was showing a higher exacerbation during the rainy season.

17.8% (102) of the subjects had loss of sleep due to on and off allergic lesions such as itching and rashes in the past 12 months, but the frequency was less than once a week. The overall history of itching, dermatitis in the past was seen in 24.8% (142) of the subjects.

We found a significant association between history of itching, rashes, dermatitis and wheezing (*P* < 0.0001). In this, 23 subjects had wheeze among 48 subjects with itching, rashes in the past ever. Also there was a statistically significant association between hay fever and wheezing (*P* < 0.0001). This showed that the subjects with history of itching, rashes, dermatitis, hay fever are more likely to be wheezers as compared to those without this history.

In Kerala, Ravindran P *et al*,[5] studied the overall prevalence of asthma by dividing the subjects as those having wheezing at the time of survey and were on medication (current wheezers) and those gave a history of wheeze but were not suffering at the time of survey (Ever wheezed). There was overall prevalence of 5.2% to 6.1% of current wheezers and 10.2% to 13.8% of ever wheezed patients. Paramesh H *et al.*,[3] in Bangalore did a hospital based study on 20,000 children under the age of 18 years from 1979,1984,1989,1994 and 1999 in the city of Bangalore, and showed a prevalence of 9%, 10.5%,18.5%, 24.5% and 29.5% respectively. The increased prevalence correlated well with demographic changes of the city. Further to the hospital study, a school survey in 12 schools on 6550 children in the age group of 6 to 15 years was undertaken for prevalence of asthma; Children from schools of heavy traffic area showed prevalence of 19.34%; Children from low traffic area school had 11.15% respectively (*P* < 0.001). A continuation of study in rural areas showed 5.7% in children of 6–15 years. The persistent asthma also showed an increase from 20% to 27.5% and persistent severe asthma 4% to 6.5% in 1994.

Gupta D *et al*,[6] in Chandigarh, studied the prevalence of asthma and its association with environmental factors, where males had more prevalence than females. This study showed a high prevalence of asthma among the school children in a rural area. Also there was gender difference in the symptoms and prevalence of asthma and allergic rhinitis. This calls for further evaluation of genetic and environmental factors which determine the prevalence, exacerbations of these allergic diseases. The environmental determinants of the allergic diseases among the children can be studied, compared to the national and international studies. This would also guide for the future measurement of the trend in the disease over a period of time.
